# A nomogram for predicting small bowel mucosal healing in pediatric Crohn’s disease

**DOI:** 10.3389/fmed.2025.1582238

**Published:** 2025-06-24

**Authors:** Bingxia Chen, Huiwen Li, Hongli Wang, Lu Ren, Liya Xiong, Yang Cheng, Rui Li, Meiwan Cao, Zihuan Zeng, Sitang Gong, Peiyu Chen, Lanlan Geng

**Affiliations:** Guangzhou Women and Children’s Medical Center, Guangzhou Medical University, Guangzhou, China

**Keywords:** Crohn’s disease, capsule endoscopy, mucosal healing, nomogram, prediction model

## Abstract

**Objectives:**

According to the updated Selecting Therapeutic Targets in Inflammatory Bowel Disease (STRIDE-II), mucosal healing (MH) is the long-term therapeutic target for Crohn’s disease (CD). Capsule endoscopy (CE) is effective in evaluating small bowel mucosal inflammation. This research seeks to construct a simple tool for predicting small bowel MH in pediatric CD to aid clinical decision-making.

**Methods:**

Data from the medical records of patients with CD who underwent CE at the Guangzhou Women and Children’s Medical Center between November 2017 and July 2022 were retrospectively analyzed. The least absolute shrinkage and selection operator (LASSO) logistic regression algorithm was applied to identify predictive factors for small bowel MH. A nomogram incorporating these factors was constructed to predict the probability of MH in this population.

**Results:**

In total, 143 CE examinations performed in 91 pediatric CD patients (median age, 11 years) were included. Based on the Lewis scores, the CD patients were divided into “MH” (42 cases) and “non-MH” groups (101 cases). LASSO regression analysis identified erythrocyte sedimentation rate, albumin levels, aspartate transaminase levels, C-reactive protein levels, platelet count, and lymphocyte percentage as the most significant predictors; and thus, these factors were incorporated into the predictive nomogram model. The area under the receiver-operating characteristic (ROC) curve of the predictive nomogram model was 0.855 (95% confidence interval, 0.783–0.926), suggesting a high discrimination power.

**Conclusion:**

A nomogram was constructed to predict small bowel MH in pediatric CD patients. This nomogram model can enable accurate and simple attentive observation of small bowel inflammation in CD patients.

## Introduction

Crohn’s disease (CD), a relapsing and remitting inflammatory disease, often requires surgery due to complications, such as strictures, fistulae, and abscesses (1). CD affects the entire gastrointestinal tract from the oral cavity to the anus. Up to 70% of CD patients present with small bowel involvement, and approximately half of the cases involve only the small bowel (2–5). Furthermore, small bowel CD is more refractory and has a higher incidence of intestinal complications than colon CD (6). Nevertheless, assessment of small bowel activity in the CD is relatively more difficult than that of the colon. And it has been reported that small bowel inflammation is considerably underestimated (7).

Mucosal healing (MH) is defined as the resolution of visible inflammation and ulceration during endoscopy, which is an important objective in the disease monitoring of patients with CD. It is widely accepted that MH can reduce the risk of bowel damage, thereby leading to improved long-term outcomes (8–12). Therefore, patients with CD need an evaluation of the entire small intestine, not only to diagnose or assess disease activity before and after treatment but also to confirm the maintenance of MH. Capsule endoscopy (CE) has been utilized to evaluate small bowel pathology in adults and children since 2001 and 2004, respectively (13). In patients with established CD, CE is used for assessing disease activity and extent and monitoring treatment effects and postoperative recurrence (14). CE scores, i.e., Niv and Lewis scores, are effective in predicting clinical outcomes in patients with CD (15–17). Moreover, CE findings can also modify the treatment plan of CD, sometimes avoiding surgery altogether (18).

Nevertheless, despite its obvious advantages, CE has a retention risk, is costly, and requires qualified medical personnel and patient preparation. A meta-analysis reported that CE retention occurred in 3.49% and 1.64% of adult and pediatric CD, respectively (19). The retention rate could be reduced to 1.3% with the use of patency capsule (20). Predicting the rate of small bowel MH can optimize individual treatment strategies timely when CE is unavailable.

Currently, accurate instruments with high sensitivity and specificity that use serological parameters and clinical characteristics to predict MH evaluated by CE in pediatric CD are lacking. Therefore, we aimed to use routine clinical and serological data to construct a nomogram for predicting MH evaluated by CE in pediatric CD.

## Materials and methods

### Study population

This retrospective study was conducted at the Guangzhou Women and Children’s Medical Center and included consecutive patients aged 1–17 years with established CD who underwent CE between November 2017 and July 2022. Patients without small bowel involvement were excluded from the analysis. The subjects whose CE did not reach the colon during the recording time and those who lacked clinical or laboratory indices were also excluded. The CD diagnosis was retrospectively confirmed based on the Porto criteria defined by the European Society for Paediatric Gastroenterology Hepatology and Nutrition (ESPGHAN) (21). Data on patients’ baseline information and that corresponding to the point of CE examination, including age, sex, the Paris Classification (22), weighted Pediatric Crohn’s Disease Activity Index (wPCDAI) (23), and serological parameters, were collected.

### CE procedure

Capsule endoscopy (MiroCam, South Korea) was performed after bowel preparation with polyethylene glycol or mannitol and overnight fasting. If a patient was not able to swallow the CE, a gastroscope was used to deliver the CE. Mucosal inflammation was evaluated using the Lewis scores and MH was defined as a Lewis score of <135 (24).

### Construction and evaluation of predictive models

The least absolute shrinkage and selection operator (LASSO) logistic regression algorithm was applied to identify the most important predictors, minimizing excessive fitting or selection bias in basic features. Subsequently, these variables were used to construct a nomogram. The area under the receiver-operating characteristic curve (AUC) was calculated to assess the nomogram’s discriminative ability, which was then corrected by bootstrapping validation. Furthermore, the calibration curve was presented to illustrate the relationship between predicted probabilities and observed frequencies.

### Statistical methods

R 4.2.0 software was used to perform statistical analysis. Normally distributed continuous variables were reported as means ± SD and compared using a 2-sample unpaired *t*-test. Non-normally distributed continuous variables were presented as medians and interquartile range (IQR) and compared using the Mann–Whitney *U* test. Categorical variables were summarized as counts and percentages and analyzed by Fisher’s exact test or Chi-squared test. The “glmnet” package was used to select the most significant predictors. The “regplot” and “rms” packages were used to construct the nomogram and presented calibration curves. Receiver-operating characteristic (ROC) curves were plotted using the “pROC” package. A *P-*value of <0.05 was considered statistically significant.

## Results

### Dataset characteristics

Overall, 203 CE examinations were performed in patients with CD at the Guangzhou Women and Children’s Medical Center between November 2017 and July 2022. A total of 12 cases with incompleteness of CE and 48 cases with data defects were excluded from the analysis. Thus, 143 CE examinations performed in 91 patients with CD were included in this study (median age, 11 years). Based on the Lewis scores, the patients with CD were divided into “MH” and “non-MH” groups. A total of 42 cases achieved MH. Table 1 shows the clinical and demographic characteristics of the included cases. The comparison of demographics and laboratory test results between the MH and non-MH groups is presented in Table 2.

**TABLE 1:** Characteristics of included cases.

Characteristics	Median (IQR) or *n* (%)
Male (*n*, %)	87 (60.8%)
Age, years	11 (8–13)
Disease location	
L1	3 (2.0%)
L1 + L4a	1 (0.6%)
L1 + L4b	20 (14.0%)
L1 + L4a + b	7 (4.9%)
L2 + L4b	19 (13.3%)
L3	5 (3.5%)
L3 + L4b	58 (40.6%)
L3 + L4a + b	10 (7.0%)
L4b	14 (9.8%)
L4a + b	6 (4.2%)
Behavior	
B1	139 (97.2%)
B2	4 (2.8%)
B3	0
Linear growth failure	5 (3.5%)
Perianal disease	13 (9.1%)
Current treatment	
Anti-tumor necrosis factor-α therapy	53 (37.1%)
Anti-tumor necrosis factor-α therapy + thiopurines	1 (0.7%)
EEN	20 (14.0%)
Corticosteroids	1 (0.7%)
Thiopurines	1 (0.7%)
MTX	4 (2.8%)
Thalidomide	1 (0.7%)
None	62 (43.4%)

Categorical data were presented as frequency (percentage), continuous data were presented as median (interquartile ranges). EEN, exclusive enteral nutrition; MTX, methotrexate.

**TABLE 2:** Differences between small bowel MH and non-MH in pediatric CD patients.

Characteristics	MH (*n* = 42)	Non-MH (*n* = 101)	*P-*value
Sex (M/F)	28/14	59/42	0.357
Age (years)	10.31 (7.40–13.22)	10.68 (7.65–13.71)	0.498
wPCDAI	7.5 (0–14.4)	27.5 (10.0–47.5)	<0.001
ESR (mm/h)	20.62 (2.59–38.65)	44.30 (14.19–74.40)	<0.001
ALB (g/L)	43.57 (39.50–47.64)	37.53 (30.22–44.85)	<0.001
GLO (g/L)	30.04 (24.97–35.11)	32.31 (27.18–37.44)	0.017
TBA (μmol/L)	3.10 (2.55–5.95)	3.95 (2.20–6.30)	0.838
TB (μmol/L)	5.61 (1.45–9.78)	4.21 (1.41–7.02)	0.050
DB (μmol/L)	1.26 (0.20–2.32)	1.14 (0.40–1.88)	0.496
IB (μmol/L)	4.36 (1.03–7.70)	3.08 (0.85–5.30)	0.026
ALT (U/L)	13 (12–18)	8 (6–13)	<0.001
AST (U/L)	23 (20–25)	18 (14–22)	<0.001
GGT (U/L)	12.45 (7.34–17.56)	14.22 (6.54–21.89)	0.111
ALP (U/L)	209.93 (132.96–286.90)	168.10 (91.16–245.04)	0.036
LDH (U/L)	192.79 (156.42–229.16)	174.01 (126.67–221.35)	0.023
Cr (μmol/L)	40.50 (27.17–53.83)	40.42 (27.37–53.46)	0.972
CK (U/L)	108.00 (81.50–130.75)	57.00 (34.00–84.00)	<0.001
CRP (mg/L)	0.92 (0.49–2.75)	20.75 (2.46–49.46)	<0.001
WBC (10^9^/L)	6.80 (5.70–8.48)	8.85 (7.30–11.70)	<0.001
PLT (10^9^/L)	331.71 (226.70–436.72)	464.23 (321.25–607.20)	<0.001
HCT (%)	37.72 (33.80–41.64)	35.44 (30.83–40.04)	0.006
Neutrophil (%)	50.00 (41.25–64.25)	62.00 (54.00–69.00)	<0.001
Lymphocyte (%)	38.00 (25.25–44.50)	25.00 (19.00–33.00)	<0.001
Monocyte (%)	6.00 (6.00–9.00)	7.00 (6.00–10.00)	0.009
Eosinophil (%)	2.00 (1.00–5.00)	2.00 (1.00–4.00)	0.518
Basophil (%)	0.00 (0.00–1.00)	0.00 (0.00–1.00)	0.173

Values are expressed as the mean ± SD or median (IQR). MH, mucosal healing; non-MH, non-mucosal healing; wPCDAI, weighted Pediatric Crohn’s Disease Activity Index; ESR, erythrocyte sedimentation rate; ALB, albumin; GLO, globulin; TBA, total bile acid; TB, total bilirubin; DB, direct bilirubin; IB, indirect bilirubin; ALT, alanine aminotransferase; AST, aspartate aminotransferase; GGT, gamma-glutamyltransferase; ALP, alkaline phosphatase; LDH, lactic dehydrogenase; Cr, creatinine; CK, creatine kinase; CRP, C-reactive protein; WBC, white blood cells; PLT, platelet; HCT, hematocrit.

### Feature selection

**FIGURE 1:**
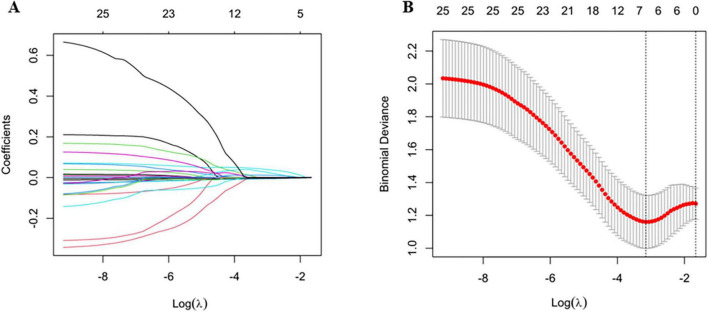
Screening of variables based on LASSO regression. **(A)** The variation characteristics of the coefficient of variables; **(B)** the selection process of the optimum value of the parameter λ in the LASSO regression model by 10-fold cross-validation method.

The LASSO logistic regression algorithm was applied to select the most significant predictors. The 10-fold cross-validation method was applied to the iterative analysis, and a model with good performance but minimum number of variables was obtained when λ was 0.043 (Log λ = −3.15) (Figure 1). Among 26 clinical features analyzed in the LASSO logistic regression analysis, 6 features with non-zero coefficients were subsequently selected, including erythrocyte sedimentation rate (ESR), albumin (ALB), aspartate aminotransferase (AST), C-reactive protein (CRP), platelet (PLT), and lymphocyte percentage. The results of multivariate logistic regression analysis of the above features are shown in Table 3.

**TABLE 3:** Multivariate logistic regression model for the prediction of small bowel MH in pediatric CD.

Valuables	OR (95% CI)	*P*-value
ESR	0.991 (0.962–1.017)	0.516
ALB	1.068 (0.963–1.170)	0.163
AST	1.071 (0.998–1.158)	0.070
CRP	0.987 (0.959–1.007)	0.261
PLT	0.994 (0.988–0.999)	0.026
Lymphocyte	1.012 (0.966–1.060)	0.623

OR, odds ratio; CI, confidence interval; ESR, erythrocyte sedimentation rate; ALB, albumin; AST, aspartate aminotransferase; CRP, C-reactive protein; PLT, platelet.

### Nomogram construction

**FIGURE 2:**
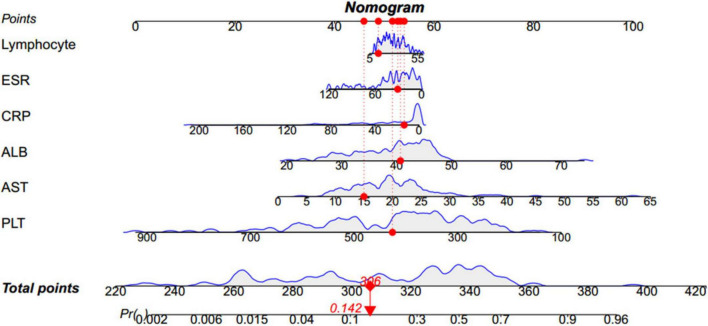
Nomogram to predict the rate of small bowel MH. For instance, for a child whose conditions are the following: 14% lymphocyte, 31 mm/h ESR, 13.43 mg/L CRP, 40.7 g/L ALB, 15 U/L AST, 426 × 10^9^/L PLT, the nomogram provides a total score of 306 points and the probability of small bowel MH is 0.142, as shown in this figure. MH, mucosal healing; ESR, erythrocyte sedimentation rate; CRP, C-reactive protein; ALB, albumin; AST, aspartate aminotransferase; PLT, platelet.

**FIGURE 3:**
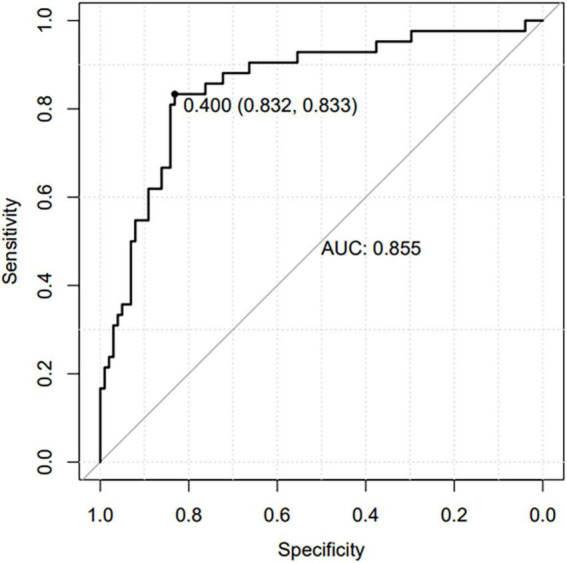
Receiver operating characteristic (ROC) curve of the nomogram prediction model.

**FIGURE 4:**
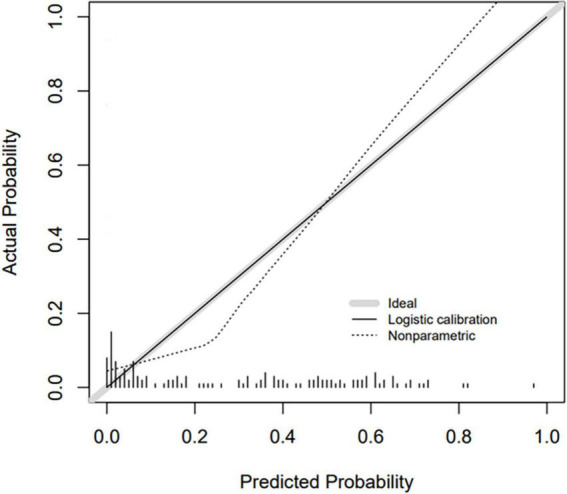
Calibration curve for nomogram prediction model.

Based on the β coefficients of the identified features in multivariate logistic regression model, the predicted rate of small bowel MH was calculated as Logit (*P*) = −2.5576–0.0093 × ESR (mm/h) + 0.0658 × ALB (g/L) + 0.0687 × AST (U/L) − 0.0132 × CRP (mg/L) − 0.0062 × PLT (10^9^/L) + 0.0115 × Lymphocyte (%). For easier clinical application, we built a nomogram to predict the rate of small bowel MH (Figure 2). The total points were calculated by adding the score in each row of variables. The AUC of this predictive nomogram model was 0.855 (95% confidence interval, 0.783–0.926) (Figure 3), and confirmed to be 0.821 through 1,000 bootstrap resamples, indicating good accuracy. The calibration plot was presented to illustrate the agreement between the observed outcome and the predicted outcome (Figure 4). The calibration curve in this study showed notable agreement between the predicted small bowel MH probability and observed MH rate as both the bias-corrected curve and the apparent curve deviated only slightly from the reference line.

## Discussion

In this study, we identified ESR, ALB, AST, CRP, PLT, and lymphocyte percentage as the most significant predictors for small bowel mucosal inflammation and used these variables to construct a nomogram to predict small bowel MH in pediatric CD patients.

Simple serological parameters and clinical characteristics correlated with endoscopic findings are highly anticipated surrogates for monitoring disease activity. Some clinical and serological parameters, including Crohn’s Disease Activity Index (CDAI), CRP levels, ESR, ALB levels, neutrophil to lymphocyte ratio (NLR), platelet to lymphocyte ratio (PLR), and CRP to ALB ratio (CAR), are reported associated with MH in patients with CD (25–27). The most widely recognized serological inflammation marker for monitoring disease activity in patients with CD is CRP (25, 26, 28). As an acute phase protein, serum CRP testing is widely available and relatively inexpensive to obtain. Increased CRP levels indicate mucosal inflammation and a probability of clinical relapse (29). However, elevated CRP levels might be related to other inflammatory disorders like infectious diseases. ESR testing is helpful to evaluate chronic and subacute inflammation and used as an aid to CRP. Hu et al. (25) found that the CD patients in endoscopic remission had significantly lower CRP, neutrophils, PLT, and higher hemoglobin (HB), lymphocytes, ALB than those with endoscopic activity. Huang et al. (30) reported agreement between the Simple Endoscopic Score for CD (SES-CD) scores and hs-CRP (*r* = 0.313, *P* < 0.001), ESR (*r* = 0.298, *P* = 0.001), white blood cells (WBCs) (*r* = 0.258, *P* = 0.005), fibrinogen (FIB) (*r* = 0.234, *P* = 0.013), plateletcrit (PCT) (*r* = 0.357, *P* < 0.001), and PLT (*r* = 0.303, *P* = 0.001). PLT is derived from bone marrow megakaryocytes and participates in wound repair and tissue regeneration. An increased PLT indicates an inflammatory activation state since PLTs release various bioactive inflammatory particles and express various inflammatory receptors under chronic inflammatory conditions (31, 32). Lymphocyte level was reported to decrease during the active period in pediatric patients with inflammatory bowel disease (IBD) due to an increase in whole-blood lymphocyte apoptosis (33). On the other hand, a relative increase of neutrophil percentage contributed to the relative decrease of lymphocyte percentage. A retrospective study reported that the severity of endoscopic activity had a positive correlation with PLT (*r* = 0.458, *P* < 0.001) and a negative correlation with lymphocyte percentage (34). ALB, a negative acute phase reactant, decreased during intestinal inflammation, attributing to malnutrition and malabsorption (35). AST is a liver function biomarker and no study has explored the relationship between AST level and CD activity.

Numerous studies have investigated predictive models using non-invasive biomarkers for the assessment of MH in CD. In 2015, Minderhoud et al. (36) reported an index predicting endoscopic disease activity in patients with CD based on clinical and laboratory parameters. Recently, D’Haens (37) proposed a novel serum-based assay for mucosal inflammation in CD, the endoscopic healing index (EHI), which identified patients with resolved endoscopic disease activity with good overall accuracy. Nevertheless, most of them used ileocolonoscopy to determine the MH in CD, which cannot evaluate the entire small intestinal mucosa.

Mucosal healing in the small bowel and colon are not always synchronous. Takenaka et al. (38) evaluated the endoscopic healing of different site involvement among patients who received anti-TNF-α treatment in a *post hoc* analysis. They reported that small bowel and colonic endoscopic healing was achieved in 36% (41/114) and 79% (33/42) cases during maintenance therapy, with a significant difference (38). Studies comparing CD patients with and without small bowel involvement have reported CD patients with small bowel involvement were more serious than those with colonic involvement and needed more aggressive treatment (39–41). The incorporation of CE to assess MH in treat-to-target algorithms for CD when the small bowel is involved has been proposed, considering the higher incidence of intestinal complications in small bowel CD (15, 16).

A few studies have explored the relationship between simple biomarkers and small bowel MH evaluated by CE. Yang et al. (42) showed that the correlation between Lewis scores and CRP levels was moderate (*r* = 0.58, *P* < 0.01). He et al. (43) analyzed 150 patients with CD (including 30 children and adolescents) who underwent CE and reported weak correlations between the Lewis scores and CRP levels in pediatric patients (*r* = 0.379, *P* = 0.044). Mitselos et al. (44) reported that CDAI and CRP levels correlated with Lewis scores and the AUC toward endoscopic activity prediction was 0.70 and 0.69, respectively. However, most of the studies focused on adult CD. In our study, ESR, ALB, AST, CRP, PLT, and lymphocyte percentage were identified as important variables and utilized to construct a nomogram for predicting small bowel MH in pediatric CD, which may monitor response to the therapy and provide early warnings of relapse.

As far as we know, this is the first study to investigate the nomogram using serological parameters and clinical characteristics to distinguish between small bowel MH and non-MH in pediatric CD patients. The predictive model demonstrated superior performance, suggesting its high value in aiding clinical decision-making.

Notably, this study has a few limitations. Firstly, due to the retrospective approach, selection bias and missing data were inevitable. It is frustrating that not all patients had fecal calprotectin test and radiological imaging (computed tomography enterography and magnetic resonance enterography) at the time of CE examination because these tests were costly and inconvenient; therefore, the evaluation of these factors as predictors in our model was limited. Secondly, the amount of data used in this study was small and it is necessary to expand the amount of data used in order to improve the model’s learning ability and reduce its bias. Thirdly, we lacked external validation. In the future, prospective, multicenter validation using large-scale studies are needed to confirm these results.

## Conclusion

In conclusion, the model described in this study is feasible for differentiating between pediatric CD patients with small bowel MH and those with active lesions. Using readily available parameters, the model can provide early warnings for actionable feedback, which may improve the outcome of pediatric patients with CD in clinical settings.

## Data Availability

The raw data supporting the conclusions of this article will be made available by the authors, without undue reservation.
